# On the Use of Etherial Solution of Gun Cotton in the Cure of Erectile Tumors without Operation

**Published:** 1849-11

**Authors:** Daniel Brainard

**Affiliations:** Prof. of Surgery in Rush Medical College, Chicago


*On the use of Etherial Solution of Gun Cotton in the cure of Erectile Tumors without Operation.* By Daniel Brainard, M. D., Prof. of Surgery in Rush Medical College, Chicago.—This adhesive liquid which was ushered into the profession with great recommendations as a substitute for needles in cases of hare lip, and for adhesive plaster in wounds, seems to have failed in fulfilling the expectations which were excited of its usefulness, and to have become rather an article of the *toilette*, and a substitute for court plaster, than a useful addition to our surgical armory. Struck, however, in the experiments with it, with the contractile power it possesses, I determined to test its application to the surface of any erectile tumor which might present itself for treatment.

During the last winter a case of nævus of the size of a very large strawberry, situated on the anterior fontanelle of a young infant, was presented for operation. I immediately covered it with a solution of gun cotton, and, although it was much elevated above the surface, had the satisfaction of seeing it brought by the contractile power of the liquid in drying to a level with the sound skin. It was allowed to remain for several weeks, and then a fresh application made; and at the present time scarcely any trace of the nævus remains, although but two applications have been made.

The next case was that of a young child, with a nævus three fourths of an inch in length, and half an inch in breadth, situated beneath the right eye. This at birth was scarcely perceptible; but in six moths had acquired the size mentioned, and was rapidly increasing. In order to avoid the irritation resulting from its proximity to the eye, the application was made during the sleep of the infant, and was required to be renewed twice a week, on account of its becoming loosened. After two months use, the nævus is scarcely perceptible, and the use of the solution has been for sometime discontinued.

It is not improbable, that by preventing the necessity of resorting to operations in such cases, this liquid may find a use more important than any to which it has before been applied.—*North-western Med. and Sur. Journal.*

